# Evaluation and development of deep neural networks for RNA 5-Methyluridine classifications using autoBioSeqpy

**DOI:** 10.3389/fmicb.2023.1175925

**Published:** 2023-05-18

**Authors:** Lezheng Yu, Yonglin Zhang, Li Xue, Fengjuan Liu, Runyu Jing, Jiesi Luo

**Affiliations:** ^1^School of Chemistry and Materials Science, Guizhou Education University, Guiyang, China; ^2^Department of Pharmacy, Affiliated Hospital of North Sichuan Medical College, Nanchong, China; ^3^School of Public Health, Southwest Medical University, Luzhou, China; ^4^School of Geography and Resources, Guizhou Education University, Guiyang, China; ^5^School of Cyber Science and Engineering, Sichuan University, Chengdu, China; ^6^Basic Medical College, Southwest Medical University, Luzhou, China; ^7^Sichuan Key Medical Laboratory of New Drug Discovery and Druggability Evaluation, Luzhou Key Laboratory of Activity Screening and Druggability Evaluation for Chinese Materia Medica, Southwest Medical University, Luzhou, China

**Keywords:** RNA 5-methyluridine, deep learning, autoBioSeqpy, UMAP, SHAP

## Abstract

Post-transcriptionally RNA modifications, also known as the epitranscriptome, play crucial roles in the regulation of gene expression during development. Recently, deep learning (DL) has been employed for RNA modification site prediction and has shown promising results. However, due to the lack of relevant studies, it is unclear which DL architecture is best suited for some pyrimidine modifications, such as 5-methyluridine (m^5^U). To fill this knowledge gap, we first performed a comparative evaluation of various commonly used DL models for epigenetic studies with the help of autoBioSeqpy. We identified optimal architectural variations for m^5^U site classification, optimizing the layer depth and neuron width. Second, we used this knowledge to develop Deepm5U, an improved convolutional-recurrent neural network that accurately predicts m^5^U sites from RNA sequences. We successfully applied Deepm5U to transcriptomewide m^5^U profiling data across different sequencing technologies and cell types. Third, we showed that the techniques for interpreting deep neural networks, including LayerUMAP and DeepSHAP, can provide important insights into the internal operation and behavior of models. Overall, we offered practical guidance for the development, benchmark, and analysis of deep learning models when designing new algorithms for RNA modifications.

## Introduction

To date, over 170 types of chemical modifications have been identified in cellular RNAs, which contain not only some common types such as N^6^-methyladenosine (m^6^A), 5-methylcytosine (m^5^C), N^1^-methyladenosine (m^1^A), pseudouridine (*Ψ*), 5-hydroxymethylcytosine (5hmC), and 2’-O-methylation of ribose (2’-O-Me), but also several rare types, including 7-methylguanosine (m^7^G), adenosine-to-inosine (A-to-I), dihydrouridine (D), N^2^-methylguanosine (m^2^G), and N4-acetylcytidine (ac4C; El Allali et al., 2021). All four RNA bases, as well as the ribose sugar, can be the targets for modification, and almost all RNA species are modified, with transfer RNA (tRNA) and ribosomal RNA (rRNA) being the most heavily modified (Roundtree et al., 2017; Chen et al., 2020). RNA modifications affect numerous biological processes, including regulation of post-transcriptional gene expression, mRNA life cycle, RNA localization, ncRNA biogenesis and function (Alarcón et al., 2015; Meyer et al., 2015; Nachtergaele and He, 2018). Accordingly, aberrant modifications are widely lined to developmental disease (Jonkhout et al., 2017). Increasing evidence suggests that RNA modification pathways are also misregulated in cancers and may be ideal targets for cancer therapy (Delaunay and Frye, 2019; Barbieri and Kouzarides, 2020).

Recent advances in studying RNA modifications have been benefited tremendously from improved detection methods. Liquid chromatography coupled with mass spectrometry (LC–MS) and next-generation sequencing (NGS) are two main methodologies for identifying and quantifying RNA modifications (Wiener and Schwartz, 2021). The LC–MS allows direct measurement of many modifications with excellent sensitivity and specificity, but is limited in its ability to determine sequence context (Su et al., 2014; Wetzel and Limbach, 2016). In contrast to LC–MS, high-throughput sequencing can provide information about the sequence context of long RNAs, which facilitates the detection of modifications in a transcriptome-wide manner (Li et al., 2016). However, most RNA modifications cannot be directly detected by NGS-based approaches, because all RNA-sequencing NGS library generation protocols include a reverse transcription step where RNA is converted into DNA (Sarkar et al., 2021). This step is sensitive to specific RNA modifications that can slow down or block the reverse transcriptase or induce the misbinding of nucleotides in the cDNA (Suzuki et al., 2015).

Owing to the high cost and technical challenge of experimentally detecting all possible modification candidates, researchers have attempted to computationally identify the epitranscriptome. Most modern computational approaches use machine learning (ML) algorithms based on handcrafted features to train a predictive model (Zhou et al., 2016; Chen et al., 2019). Although such models seem to be more transparent and controllable in construction, the bias of user assumptions in feature engineering limits their performance. In keeping with the general trend in artificial intelligence (AI), there has been a switch from classical machine learning to deep learning in newly developed RNA modification predictors. For instance, the m^6^A site predictors DeepM6ASeq (Zhang and Hamada, 2018), PM6ACNN (Alam et al., 2020), and DNN-m6A (Zhang et al., 2021b); Ψ site predictors iPseU-CNN (Tahir et al., 2019a), MU-PseUDeep (Khan et al., 2020), and PseUdeep (Zhuang et al., 2021); 5hmC site predictor iRhm5CNN (Ali et al., 2021); 2’-O-Me site predictors Deep-2’-O-Me (Mostavi et al., 2018), iRNA-PseKNC (2methyl) (Tahir et al., 2019b), and DeepOMe (Li et al., 2021); ac4C site predictors DeepAc4C and CNNLSTMac4CPred (Wang et al., 2021; Zhang et al., 2022); and a disease-associated m^7^G site predictor HN-CNN (Zhang et al., 2021a). A strength of these predictors is that they can learn modification determinants directly from sequencing data, avoiding biased user-defined features. Thus, many DL methods outperform classical ML approaches in benchmarks with different RNA modifications (Tahir et al., 2019a; Wang et al., 2021). Despite its successes, deep learning also poses challenges and limitations. First, the accessibility remains riddled with technical challenges for the nonexpert users. As most DL methods are available as source code, running them proficiently requires advanced knowledge specific to the field. Second, due to the complexity of network architectures and large training parameters, DL models are often treated as black boxes that simply mapping a given input to a model output without the explanation of how and why they work.

As another critical and abundant epigenetic mark, the 5-methyluridine (m^5^U) modification has attracted the attention of researchers worldwide. This modification is not only frequently detected in cytosolic tRNAs (Carter et al., 2019; Powell and Minczuk, 2020), but also found in other non-coding RNAs such as mRNA and rRNA (Phizicky and Alfonzo, 2010; Keffer-Wilkes et al., 2020). Some typical enzymes are involved in the catalytic procedure of m^5^U modification in different organisms, including RlmC, RlmD, and TrmA in *Escherichia coli* (Urbonavicius et al., 2007; Powell and Minczuk, 2020), Trm2 in *Saccharomyces cerevisiae* (Nordlund et al., 2000), and TRMT2A and TRMT2B (Sequence homology to TrmA and Trm2 respectively) in mammals (Carter et al., 2019; Jiang et al., 2020; Pereira et al., 2021). For this modification, the conserved T-loop motif has been found in various RNAs, which plays an important role in stabilizing the tertiary structure of RNAs (Powell and Minczuk, 2020; Pereira et al., 2021). To clarify its biological functions and understand the relevant biological processes, there is an urgent need to accurately identify RNA m^5^U sites.

Some experimental and computational methods have been developed for this mark, such as FICC-Seq, miCLIP-Seq, m5UPred and RNA-m5U (Carter et al., 2019; Jiang et al., 2020; Feng and Chen, 2022). More recently, the RNA domain separation network (RNADSN) has been proposed to abstract common features between tRNA and mRNA m5U modifications to improve the prediction of m5U sites, which mixes several layers from the convolutional neural network (CNN) and long short-term memory (LSTM; Li et al., 2022). However, studies on the identification and functional characterization of m^5^U remain limited and unexplored in current literature, and a further study on the application of deep learning in m^5^U prediction is still very necessary and useful.

In the present study, we explore the use of state-of-art DL algorithms and advanced interpretable techniques, and propose a novel computational tool for rapidly and accurately identifying RNA m5U sites. In ordor to save calculation time and make direct comparisons, only the one-hot encoding method was utilized to code RNA sequences here. Five different DL architectures such as the convolutional neural network (CNN), recurrent neural networks (RNNs) with bidirectional long short-term memory (BiLSTM) or bidirectional gated recurrent units (BiGRU), and the combination of the two networks (CNN-BiLSTM and CNN-BiGRU), were employed to build the DL models. Experimental results showed that the CNN-BiLSTM model achieved the best overall prediction performance on both of the *Full_train* and *Full_test* datasets, providing the highest scores of *ACC* (92.32 and 92.91%), and *MCC* (0.8465 and 0.8584). When performing on the cross-cell-type and cross-technique validation, the CNN-BiLSTM model also obtained satisfactory prediction results, and was eventually named Deepm5U. Using the same datasets, the predictive performance of Deepm5U was superior to that of the exiting method. Furthermore, our Deepm5U was visualized to understand how the model is processing information and making decisions. Finally, we used autoBioSeqpy (Jing et al., 2020) to develop, train, and assess different DL models, and offered a step-by-step guide on how to execute them.

## Materials and methods

### Benchmark datasets

A high-quality dataset is essential for developing a reliable prediction model. Currently, there are several public databases focused on RNA modifications, including versatile database for multiple modification types, such as RMBase (Sun et al., 2016; Xuan et al., 2018), MODOMICS (Boccaletto et al., 2018, 2022), and RMVar (Luo et al., 2021), and specialized database for a particular modification type, like Met-DB (Liu et al., 2015, 2018, 2021), REPIC (Liu et al., 2020), m^6^A-Atlas (Tang et al., 2021), CVm6A (Han et al., 2019), m6AVar (Zheng et al., 2018), m5C-Atlas (Ma et al., 2022) and m7GHub (Song et al., 2020). Unfortunately, until now, there has not been such a database available for m^5^U data. Therefore, we chose a published benchmark dataset constructed by Jing et al. (2020) to develop our deep learning models. Experimentally validated m^5^U sites (positive samples) were generated by integrating the sequencing results of FICC-seq and miCLIP-seq technologies on two cell lines, HEK293 and HAP1. The same number of unmodified uridine sites (non-m^5^U, negative samples) were randomly sampled from the same transcripts of positive samples. All samples were 41 nt in length, with the modified and unmodified uridine sites located in the center of these sequences. Based on the genomic location, positive and negative m^5^U sites were further divided into two categories: full transcript mode (uridine sites located in both exonic and intronic regions) and mature mRNA mode (uridine sites only located on mature mRNA transcripts). For each mode, total sequences were split into two mutually exclusive datasets: a training dataset of ~80% of the instances used for model derivation and an independent test set of the remaining 20% used to evaluate model accuracy (Supplementary Table S1).

In addition, Jing et al. (2020) separated the benchmark dataset into eight subsets, namely HAP1_full, HEK293_full, FICC_full, miCLIP_full, HAP1_mature, HEK293_mature, FICC_mature, and miCLIP_mature, to investigate the effects of two experimental parameters, sequencing technique, and cell type, on model prediction performance. Leave-one-subset-out cross-technique and cross-cell-type validations were performed by repeating the training-test procedure iteratively such that each subset was used as the test set exactly once. For instance, when training with the HAP1_full or HAP1_mature, the performance of model was evaluated by the HEK293_full or HEK293_mature, and vice versa. Notably, all subsets were constructed with a 1:10 positive-to-negative ratio (Supplementary Table S2).

### Deep learning techniques

Deep learning, so far the most successful form of machine learning, uses a synthetic neural network architecture composed of multiple sequential layers that can be trained on input data to achieve a prediction task. The idea of deep learning is that stacks of simple layers can learn end to end, automatically discovering a higher-level representation of the original data, which is extremely powerful and flexible in a variety of relationships that they can model (Wainberg et al., 2018). Various types of network layers, such as convolution layers, pooling layers, recurrent layers, activation layers, and fully connected layers, have been proposed to support the construction of highly flexible model architectures. CNN layers use convolution operations to fuse features that are close to each other and transfer them by kernels:


(1) $$Convolution{\left(X\right)}_{ik}=\sum_{m=0}^{M-1}\sum_{n=0}^{N-1}w_{mn}^{k}x_{i+m,n}$$

where *X* is the layer input, *i* and *k* are the indices of output position and filter kernel, respectively (LeCun et al., 2015). Convolutional filter $w^{k}$ is the M×N weight matrix with *M* and *N* being the window size and input channel, respectively. Additionally, the convolution operation can be adapted to a wider range of information fusion by changing the padding size and dilation size. For sequential data, CNN can fusion the environment of each base so that the bases differ depending on their neighbors. Sometimes similar performance can be achieved using *K*-mer or sliding window operations, but using CNNs can result in lower sparsity and an editable number of channels for further processing of the data. The pooling layer usually follows the convolution layer, and its function is to downsample layer’s input by computing the maximum or average value of the features over a region. In the RNN family, the layers use each unit of the sequence to update the hidden state for learning and inference from context. In the recurrent layer, tensors are used to represent the hidden state, and each unit of the sequence will be encoded by one or more fully connected layers for updating the hidden cells. For example, the long short-term memory (LSTM) layer contains hidden states and cell states that are updated in each iteration:


(2) $$f_{t}=\sigma \left(W_{fh}h_{t-1}+W_{fx}x_{t}+b_{f}\right)$$

(3) $$i_{t}=\sigma \left(W_{ih}h_{t-1}+W_{ix}x_{t}+b_{i}\right)$$

(4) $${\tilde{C}}_{t}=\tanh\left(W_{Ch}h_{t-1}+W_{Cx}x_{t}+b_{c}\right)$$

(5) $$C_{t}=f_{t}C_{t-1}+i_{t}\times {\tilde{C}}_{t}$$

(6) $$o_{t}=\sigma \left(W_{oh}h_{t-1}+W_{ox}x_{t}+b_{o}\right)$$

(7) $$h_{t}=o_{t}\times \tanh\left(C_{t}\right)$$

Where $C_{t}$ and $h_{t}$ are the cell state and hidden state, respectively (Hochreiter and Schmidhuber, 1997; Cho et al., 2014; Jin et al., 2021). Whereas the gate recurrent unit (GRU) layer only updates the hidden state:


(8) $$r_{t}=\sigma \left(W_{rh}h_{t-1}+W_{rx}x_{t}+b_{r}\right)$$

(9) $${\tilde{h}}_{t}=\emptyset_{h}\left(W_{hh}\left(r_{t}\times h_{t-1}\right)+W_{hx}x_{t}+b_{h}\right)$$

(10) $$z_{t}=\sigma \left(W_{zh}h_{t-1}+W_{zx}x_{t}+b_{z}\right)$$

(11) $$h_{t}=\left(1-z_{t}\right)\times {\tilde{h}}_{t}+z_{t}\times h_{t-1}$$

In addition, bidirectional operation, which allows RNN layers to learn a sequence starting from both head and tail directions, since RNNs process sequences without a predetermined direction. Dense layer, also known as fully connected layer, is the simplest type of layer, where every input is connected to every output. The role of activation function is to introduce the nonlinearity in the input–output relationship. Frequently used activation functions include sigmoid and rectified linear unit (ReLU), which are given by:


(12) $$\mathrm{Sigmoid}\left(x\right)=\frac{1}{1+e^{-x}}$$

(13) $$\mathrm{ReLu}\left(x\right)=\left\{\begin{array}{c} x\;if\;x\geq 0\\ 0\;if\;x<0 \end{array}\right.$$

Usually, ReLU is used for the nonlinear gain of the model, while sigmoid is used in the output layer of the binary classification model.

### Model architectures

We designed five architectures, namely, CNN, BiLSTM, BiGRU, CNN-BiLSTM and CNN-BiGRU, which use 20 nucleotides on each side of a position of interest as input, and output the probability of the position being an m^5^U site and a non-m^5^U site. The input to the models is a sequence of one-hot encoded nucleotides, where A, C, G, and U are encoded as [1, 0, 0, 0], [0, 1, 0, 0], [0, 0, 1, 0], and [0, 0, 0, 1] respectively and the output of the models is a score in the range [0, 1], representing positive (T or 1) and negative (F or 0) classes. We run a grid search to exhaustively test the combinations of convolution layers (1, 2, 3), kernel size (3, 5, 7, 9, 11), number of filters (50, 150, 250), pool size (2, 4, 6, 8, 10), LSTM layers (1, 2, 3), number of units in the LSTM layer (32, 64, 128, 256), GRU layers (1, 2, 3), and number of units in the GRU layer (32, 64, 128, 256) to select the best hyperparameters for the models. Details about optimal hyperparameters and model architectures are provided in Supplementary Figure S1; Supplementary Tables S3–S5 and below, respectively.

*Convolutional neural network architecture (CNN).*

(1) Convolution layer (250 filters; kernel size, 11; ReLU activation; 0% dropout; step size, 1)
(2) Convolution layer (250 filters; kernel size, 11; ReLU activation; 0% dropout; step size, 1)
(3) Pooling layer (maximum value; pool size, 10, step size, 10)
(4) Fully connected layer (256 units)
(5) Dropout layer (20% dropout)
(6) Activation layer (ReLU activation)
(7) Output layer (1 units, sigmoid activation)

*Bidirectional long short-term memory architecture (BiLSTM).*

(1) Bidirectional LSTM layer (256 units, tanh activation; sigmoid recurrent activation; 0% dropout)
(2) Bidirectional LSTM layer (256 units, tanh activation; sigmoid recurrent activation; 0% dropout)
(3) Fully connected layer (256 units)
(4) Dropout layer (20% dropout)
(5) Activation layer (ReLU activation)
(6) Output layer (1 unit, sigmoid activation)

*Bidirectional gated recurrent unit architecture (BiGRU).*

(1) Bidirectional GRU layer (256 units, tanh activation; sigmoid recurrent activation; 0% dropout)
(2) Fully connected layer (256 units)
(3) Dropout layer (20% dropout)
(4) Activation layer (ReLU activation)
(5) Output layer (1 unit, sigmoid activation)

*Convolutional-bidirectional long short-term memory architecture (CNN-BiLSTM).*

(1) Convolution layer (250 filters; kernel size, 7; ReLU activation; 0% dropout; step size, 1)
(2) Convolution layer (250 filters; kernel size, 7; ReLU activation; 0% dropout; step size, 1)
(3) Pooling layer (maximum value; pool size, 4, step size, 4)
(4) Bidirectional LSTM layer (64 units, tanh activation; sigmoid recurrent activation; 0% dropout)
(5) Fully connected layer (256 units)
(6) Dropout layer (20% dropout)
(7) Activation layer (ReLU activation)
(8) Output layer (1 unit, sigmoid activation)

*Convolutional-bidirectional gated recurrent unit architecture (CNN-BiGRU).*

(1) Convolution layer (250 filters; kernel size, 11; ReLU activation; 0% dropout; step size, 1)
(2) Pooling layer (maximum value; pool size, 10, step size, 10)
(3) Bidirectional GRU layer (256 units, tanh activation; sigmoid recurrent activation; 0% dropout)
(4) Fully connected layer (256 units)
(5) Dropout layer (20% dropout)
(6) Activation layer (ReLU activation)
(7) Output layer (1 unit, sigmoid activation)

### Model training

All models were trained using the Adam optimizer with a learning rate of 0.001, epoch of 20 and batch size of 64. During training, the sequences were first re-shuffled and subsequently randomly split into training (70%), validation (10%) and testing (20%) fractions. The validation set was used to evaluate the binary cross-entropy loss after each epoch, and the test set was used to evaluate the model. For each architecture, we repeated the training procedure 5 times and used the average result of five trained models as the final prediction. To implement the models, we used the autoBioSeqpy software with Keras framework (Chollet, 2015) and trained them on a standard PC equipped an Intel Core i7-9700K CPU, 16GB working memory and a 16 GB NVIDIA GeForce RTX 2070 GPU.

### Model interpretation and visualization

We tried to interpret the DL models by visualizing the manifold of intermediate outputs and measuring the contribution of the inputs. Currently, uniform manifold approximation and projection (UMAP) library is available for projecting a high-dimensional layer into lower dimension (usually 2D for visualization) while keeping the distances of every pair of samples as possible (McInnes and Healy, 2018). To better visualize the outputs of hidden layers, we integrated the UMAP library into LayerUMAP, a new plugin for autoBioSeqpy. Using LayerUMAP, we can generate the manifold projection of any hidden layer and observe the evolution of internal representation layer by layer during the training process. Similarly, we integrated SHAP (SHapley Additive exPlanations) into autoBioSeqpy to develop DeepSHAP for measuring the contribution of sequence inputs. SHAP is an implementation of computing shapely values, which is a solution concept in game theory:


(14) $$\phi_{i}\left(f,x\right)=\underset{S\subseteq S_{all/\left\{i\right\}}}{\sum }\frac{\left|S\right|!\left(M-\left|S\right|-1\right)!}{M!}\left[f_{x}\left(S\cup \left\{i\right\}\right)-f_{x}\left(S\right)\right]=\underset{S\subseteq S_{all/\left\{i\right\}}}{\sum }\frac{1}{C_{M}^{\left|S\right|}\left(M-\left|S\right|\right)}\left[f_{x}\left(S\cup \left\{i\right\}\right)-f_{x}\left(S\right)\right]$$

where M is the number of features, S is a subset of the features, f is the model, $S_{all}/\left\{i\right\}$ is all the possible subset exclude feature i, and $f_{x}$ is the conditional expectation function. The total contribution of features can be represented by a linear combination of Shapley value:


(15) $$f\left(x\right)=\phi_{0}\left(f\right)+\overset{M}{\underset{i=1}{\sum }}\phi_{i}\left(f,x\right)$$

where $\phi_{0}\left(f\right)=f_{x}\left(\emptyset \right)$. DeepSHAP can visualize the SHAP values of input sequences using the heat maps or logo plots for the downstream analysis.

### Evaluation metrics

For evaluation, we calculated the accuracy (*ACC*), precision (*PRE*), *F*-value, recall, and Matthew’s correlation coefficient (*MCC*) as quantification metrics, which are defined as follows:


(16) $$ACC=\frac{TP+TN}{TP+FP+TN+FN}$$

(17) $$PRE=\frac{TP}{TP+FP}$$

(18) $$F-value=2\times \frac{TP}{2TP+FP+FN}$$

(19) $$Recall=\frac{TP}{TP+FN}$$

(20) $$MCC=\frac{\left(TP\times TN\right)-\left(FN\times FP\right)}{\sqrt{\left(TP+FN\right)\times \left(TN+FP\right)\times \left(TP+FP\right)\times \left(TN+FN\right)}}$$

where TP, TN, FP, and FN stand for true positive, true negative, false positive and false negative, respectively. Moreover, we plotted receiver operating characteristic (ROC) and precision-recall (PR) curves, and summarized model performance by computing areas under both ROC and PR curves, resulting in auROC and auPR, respectively.

### Overview of autoBioSeqpy

The autoBioSeqpy is a Keras-based deep learning software for fast and easy development, training, and analysis of deep learning model architectures for biological sequence classification (Jing et al., 2020). Compared with other tools or libraries, the biggest advantage of autoBioSeqpy is its simplicity and flexibility, which is especially suitable for nonexperts or users with little or no knowledge of deep learning techniques. No programming required, users only need to prepare input datasets and model templates. The operation is also simple. The entire workflows, including file reading, data encoding, parameter initialization, model training, evaluation, and visualization, can be automatically executed with just one-line instruction.

## Results

### Evaluation of representative DL models

**Figure 1:**
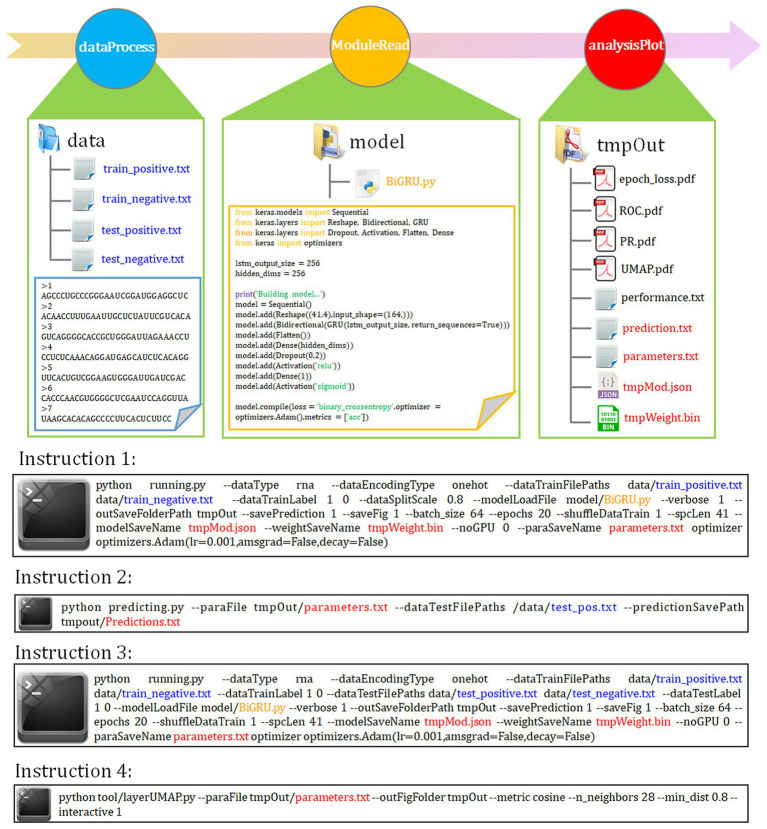
autoBioSeqpy workflow and usage. The basic framework of autoBioSeqpy consists of six modules, including four self-write modules (*ParaParser*, *dataProcess*, *moduleRead* and *analysisPlot*) and two dependent modules (*Keras* and *sklearn*). The *dataProcess* module encodes DNA, RNA, protein and compound character sequences into model-readable numerical vectors. The *moduleRead* module loads and initializes the neural network model designed by the users. The *analysisPlot* module evaluates the models on the test set. The following are some commonly used commands of autoBioSeqpy, including model training, prediction and visualization.

Five representative DL models constructed with different network architectures were used for benchmarking (“Model architectures” section). Using the instruction 1 as shown in Figure 1, we first assessed basic prediction performance of each model on full transcript mode using a stratified random sampling strategy. With the parameter “--dataSplitScale” set to 0.8, autoBioSeqpy randomly split the input sequences into 80% training-validation set and 20% test set, stratified by class. We used the shuffle mechanism (−-shuffleDataTrain 1) for each call of instruction 1 to avoid overfitting and to ensure that the model demonstrated differences. For each model, we repeated the instruction 1 five times to estimate mean and standard deviation of the seven metrics (“Evaluation metrics” section). Full results were listed and shown in Table 1; Supplementary Figure S2A, respectively. Overall, all models performed well in the intra-dataset evaluation. CNN model showed slightly worse performance compared with hybrid models, but performed better than individual RNN models. The hybrid CNN-BiLSTM model achieved the best prediction performance and provided the highest scores for *ACC* (92.32%), *F-*value (92.29%), *MCC* (0.8465), *auROC* (0.9740), and *auPR* (0.9781). CNN-BiGRU afforded the highest *Recall* score (92.46%), while CNN offered the highest *PRE* score (93.04%). In addition, an independent test set was employed to evaluate the robustness and reproducibility of the presented DL models. Here, both instruction 2 and instruction 3 can be used for this purpose, but they are suitable for different application conditions (Figure 1). For example, we can combine instruction 1 and instruction 2 to predict the probability and class of unlabelled datasets or single-labelled datasets, while for the datasets containing both positive and negative labels, we can directly call instruction 3 to generate their assessment results together with the related plots and confusion matrix. In this comparison, we see that CNN-BiLSTM still achieved the best performance, followed by CNN-BiGRU, CNN, BiGRU and BiLSTM, which was consistent with the results of the training dataset (Table 1; Supplementary Figure S2B). Next, we benchmarked the performance of above five models using the mature mRNA mode. Like the full transcript mode, no models achieved equivalent performance to CNN-BiLSTM. On average, its prediction accuracy was 91.12% for training dataset and 92.07% for independent test set (Supplementary Table S6). The ROC and PR curves were illustrated in Supplementary Figure S3.

**Table 1:** Performance comparison of different deep learning models on the full transcript mode.

Model	ACC (%)	*F*-value (%)	Recall (%)	PRE (%)	MCC	auROC	auPR
*Training dataset (Full_train)*							
CNN	91.56	91.50	90.04	**93.04**	0.8319	0.9637	0.9690
BiLSTM	88.59	88.55	87.58	89.65	0.7730	0.9419	0.9477
BiGRU	87.22	87.27	87.04	87.60	0.7820	0.9536	0.9544
**CNN-BiLSTM**	**92.32**	**92.29**	91.93	92.65	**0.8465**	**0.9740**	**0.9781**
CNN-BiGRU	91.85	91.94	**92.46**	91.44	0.8370	0.9632	0.9676
*Independent test set (Full_test)*							
CNN	91.67	91.63	91.25	92.05	0.8336	0.9689	0.9738
BiLSTM	88.39	88.53	88.60	88.47	0.7676	0.9560	0.9584
BiGRU	88.63	89.01	92.06	86.22	0.7751	0.9631	0.9646
**CNN-BiLSTM**	**92.91**	**92.96**	93.79	**92.16**	**0.8584**	**0.9773**	**0.9810**
CNN-BiGRU	91.87	92.07	**94.39**	89.95	0.8393	0.9749	0.9788

To further assess the predictive performance of CNN-BiLSTM, 41 m^5^U sites identified by Oxford Nanopore Techniques (ONT) have been collected from the DirectRMDB database (Zhang et al., 2023) as the second independent test set. As shown in supplementary Table S7, 36 of them are correctly predicted by CNN-BiLSTM, and only 5 are misclassified. Hence, CNN-BiLSTM achieves a satisfactory *ACC* (87.80%) once again. Taken together, CNN-BiLSTM constantly outperformed the other DL models and therefore was chosen as the core classification algorithm for Deepm5U, a new predictor for the m^5^U identification.

### Full view of interpretable DL models from hidden layers and input features

**Figure 2:**
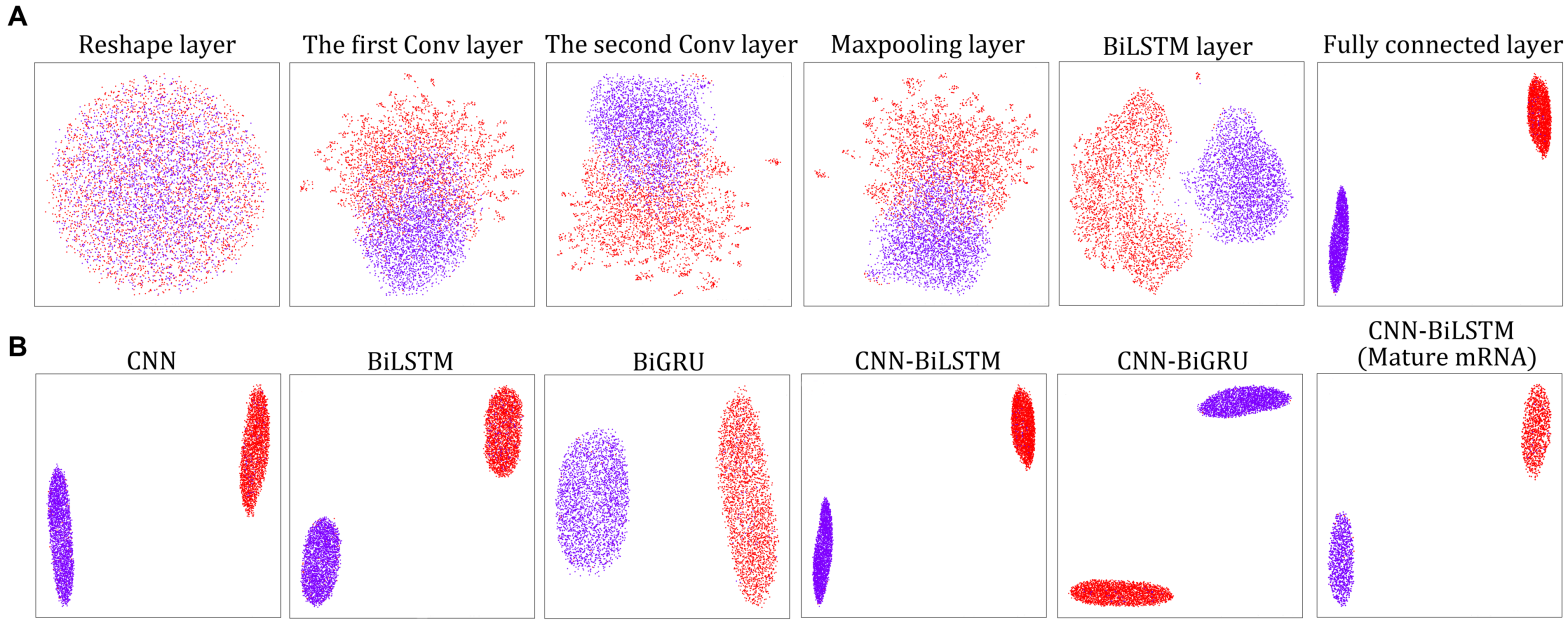
LayerUMAP reveals the inner working mechanism of the DL models. **(A)** UMAP projection of layer-to-layer evolution of the CNN-BiLSTM model for the full transcript training dataset. Colored point clouds represent m^5^U and non-m^5^U sites, showing how the model clusters the categories. **(B)** Comparison of the UMAP maps of the last hidden layer representation for other DL models and datasets.

Immediately following instruction 1 or instruction 3, we can use the layerUMAP tool to visually investigate the trained DL models via instruction 4 (Figure 1). By default, layerUMAP outputs the last hidden layer projection of the model. However, using the “--interactive” parameter, we can project any hidden layer by specifying the name or index of the layer using a list provided by layerUMAP. We first dissected the best CNN-BiLSTM model layer by layer to verify its ability to distinguish between m^5^U and non-m^5^U sites (Figure 2A). We analyzed features extracted from six hidden layers of the classification model. First, the features in the reshape layer (original one-hot encoding) were completely mixed and indistinguishable. Along the hierarchy of layers, the features became clearly distinguishable, and in the last two layers (BiLSTM layer and fully connected layer), features separated point populations into two distinct clusters according to their labels. These results demonstrated the powerful feature extraction capability of deep learning algorithm that can automatically extract the useful information from raw inputs. It is worth noting that some hidden layers, such as reshape layer, convolution layer and pooling layer, cannot be projected directly into 2D space for visualization due to their multidimensional data. Therefore, we performed dimensionality reduction on these layers to compress the multi-dimensional data into low-dimensional data from different directions. Figure 2B showed the projection of the last hidden layer for other models and datasets. Unsurprisingly, UMAP visually revealed two-point clusters that correspond to m^5^U and non-m^5^U sites, which were in line with the performance of the corresponding DL models.

**Figure 3:**
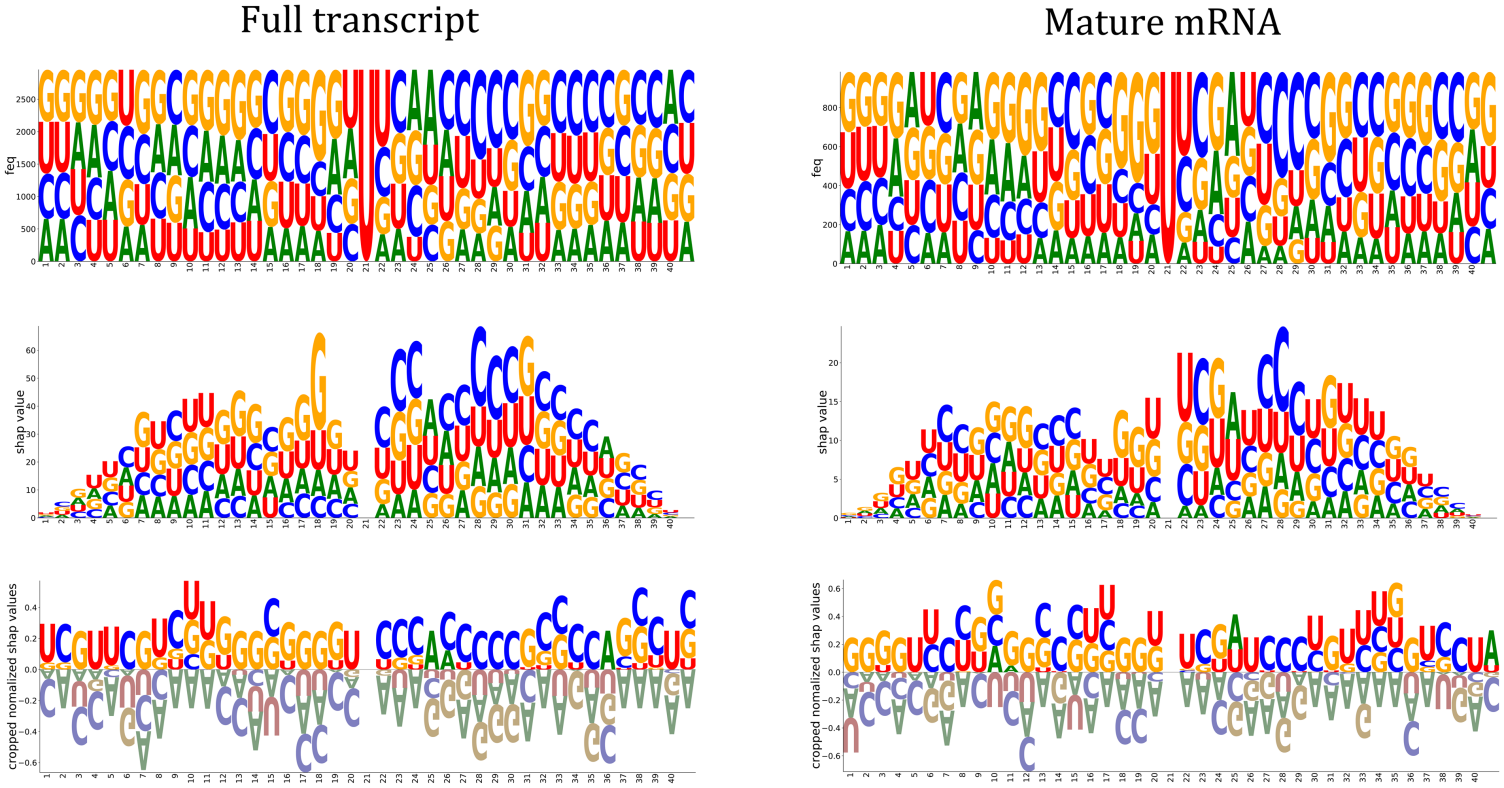
Feature importance analysis. Sequence logos representing the nucleotide composition at each position of the input sequence (Top panel). Feature importance scores associate with m^5^U identification determined by DeepSHAP (CNN-BiLSTM classifier). The height of the letter indicates the SHAP value of the relevant feature for the input sequence (Middle panel). Positive and negative normalized SHAP values represent favored and disfavored relevant features as shown in the Bottom panel.

One of the Jupyter notebook tutorials in the autoBioSeqpy demonstrated how to use DeepSHAP to measure and visualize the contribution of input sequences to a trained DL model.^1^ We used the logo plots generated by the Logomaker package instead of the commonly used summary violin plots to display the computed SHAP values (Kim et al., 2020; Tareen and Kinney, 2020). These logos allowed visualization of how important a certain nucleotide at a certain position was for the model’s classification decision. We first generated the classical sequence logos for the full-length input sequences to reveal the potential *cis*-regulatory patterns of m^5^U (Figure 3, top panel). Using 2,956 and 985 training sequences, we calculated nucleotide compositions for the full transcript mode and mature mRNA mode, respectively. We did not observe a significant difference in the motifs between the two modes. Guanine was overrepresented in the upstream region relative to the m^5^U sites, while some positions in the downstream region were enriched for cytosine. The feature importance scores associated with m^5^U identification determined by DeepSHAP were shown in the middle panel of Figure 3. Inspired by sequence logos, the height of each letter corresponded to the SHAP value of that base. Since uracil was located at the center position of all samples, its contribution to the overall prediction was zero. Nucleotides near the center contributed more to the prediction (high SHAP values), while nucleotides located on both sides had low SHAP values. GGU at positions {18–20} and CXAXCCC at positions {23–29} made a significant contribution to predicting m^5^U for both modes. This observation also coincided with the above sequence analysis. Finally, we normalized the SHAP values to highlight the favored and disfavored features. The calculated SHAP values were first scaled to the range of [−0.25, 0.25] to confirm that the summation values could lie in the range of [−1, 1], and then these values were accumulated according to the position of the base and plotted on the bottom panel of Figure 3. It was clear that adenine was a disfavored sequence feature for m5U recognition, regardless of the mode.

### Performance evaluation of Deepm5U on the cross-techniques and cross-cell-type datasets

While it is important to evaluate classification performance within a dataset (intra-dataset), realistic scenarios where classifiers are useful require to be evaluated across datasets (inter-dataset). We used eight datasets (“Benchmark datasets” section) to test the Deepm5U’ ability to predict m^5^U sites. We evaluated the classification performance when training on one dataset and testing on the other. Within intra-dataset predictions, we observed very high prediction accuracy, with *ACC* larger than 97.00% for all datasets (Table 2). However, high accuracy does not guarantee good predictive performance of model, especially with the presence of extremely unbalanced sample ratios in these datasets. We therefore examined other metrics that are more sensitive to sample imbalance, such as Recall. The average Recall of Deepm5U prediction was 87.73%, and 6 of 8 datasets were predicted with recall of at least 85.00%. The lowest Recall score was 79.90%, obtained by FICC_full dataset. These results confirmed good predictive accuracy of Deepm5U in identifying m^5^U sites. Supplementary Figure S4 showed the ROC and PR curves evaluated per sequencing protocol and per cell type for two modes. Deepm5U achieved an average auROC of 0.9841 and an auPR of 0.9507 on different classification tasks. We also visualized the positive and negative samples for all datasets using LayerUAMP based on the features learned in the last hidden layer. As shown in Supplementary Figure S5, Deepm5U mapped input sequences into different clusters according to the two-class label, and we see that the structures of red and purple classes were similar for all cases.

**Table 2:** Cross-cell-type and cross-technique validation using Deepm5U.

Mode	Testing method	Evaluation metric	Cross-technique validation		Cross-cell-type validation	
			miCLIP-Seq	FICC-Seq	HEK293	HAP1
Full transcript	Intra-dataset evaluation	*ACC* (%)	98.31	97.41	98.21	97.61
		*F*-value (%)	90.16	84.84	89.77	85.86
		*Recall* (%)	87.09	79.90	87.01	81.50
		*PRE* (%)	93.49	90.45	92.76	90.97
		*MCC*	0.8932	0.8362	0.8886	0.8478
		auROC	0.9769	0.9729	0.9858	0.9770
		auPR	0.9387	0.8975	0.9460	0.9269
	Inter-dataset evaluation	*ACC* (%)	95.64	93.90	97.10	94.33
		*F*-value (%)	71.79	54.02	82.31	58.60
		*Recall* (%)	61.05	39.44	74.29	44.13
		*PRE* (%)	87.57	85.93	92.40	87.22
		*MCC*	0.7093	0.5574	0.8135	0.5967
		auROC	0.9324	0.8332	0.9727	0.8700
		auPR	0.7958	0.6079	0.9062	0.6627
Mature mRNA	Intra-dataset evaluation	*ACC* (%)	99.36	98.30	99.38	98.22
		*F*-value (%)	96.54	89.94	96.51	90.15
		*Recall* (%)	94.62	87.80	94.89	89.04
		*PRE* (%)	98.56	92.47	98.22	91.41
		*MCC*	0.9621	0.8913	0.9619	0.8922
		auROC	0.9999	0.9820	0.9928	0.9856
		auPR	0.9998	0.9466	0.9863	0.9641
	Inter-dataset evaluation	*ACC* (%)	98.28	95.06	98.62	94.88
		*F*-value (%)	90.37	64.02	92.52	61.03
		*Recall* (%)	89.14	48.41	93.90	44.77
		*PRE* (%)	91.68	94.81	91.32	97.81
		*MCC*	0.8945	0.6574	0.9181	0.6408
		auROC	0.9931	0.8893	0.9943	0.8733
		auPR	0.9056	0.7014	0.9508	0.6951

### SHAP values explained the bias observed in cross-evaluation

**Figure 4:**
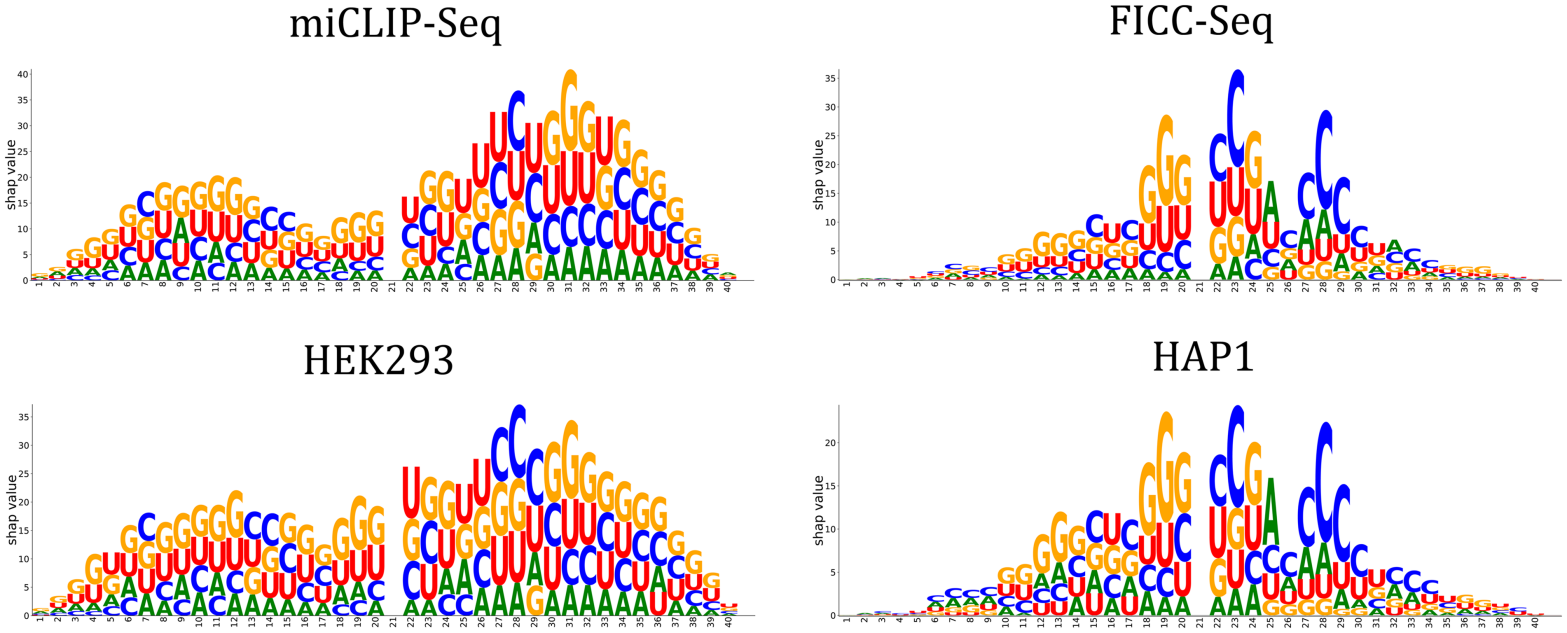
Factors affecting m5U identification for different datasets are revealed by DeepSHAP.

When evaluating the performance of Deepm5U across datasets, we found that the performance of different datasets varied greatly, and the Recall scores ranged from 39.44 to 93.90%, with a mean value of 61.89% (Table 2; Supplementary Figure S6). Closer examination of the inter-dataset evaluation revealed one interesting observation. Deepm5U models trained on miCLIP-Seq dataset or HEK293 dataset can better predict FICC-Seq dataset or HAP1 dataset, but not vice versa. The Recall score of the former (miCLIP-Seq Recall: 89.14%, HEK293 Recall: 93.90%) was nearly twice as high as that of the latter (FICC-Seq Recall: 48.41%, HAP1 Recall: 44.77%), especially for the mature mRNA mode. This phenomenon can be well explained by our DeepSHAP method (Figure 4). The distribution of SHAP values for these datasets was significantly different. The features near the central m^5^U sites played the most important role in the FICC-Seq dataset and HAP1 dataset, while for both miCLIP-Seq dataset and HEK293 dataset, features at many positions contributed to the predictions. In terms of the total SHAP values, the FICC-Seq and HAP1 datasets did not contain enough feature information to support their accurate predictions for the other two datasets.

### Variant predicting probabilities from saturation mutagenesis reveals potential valuable region

**Figure 5:**
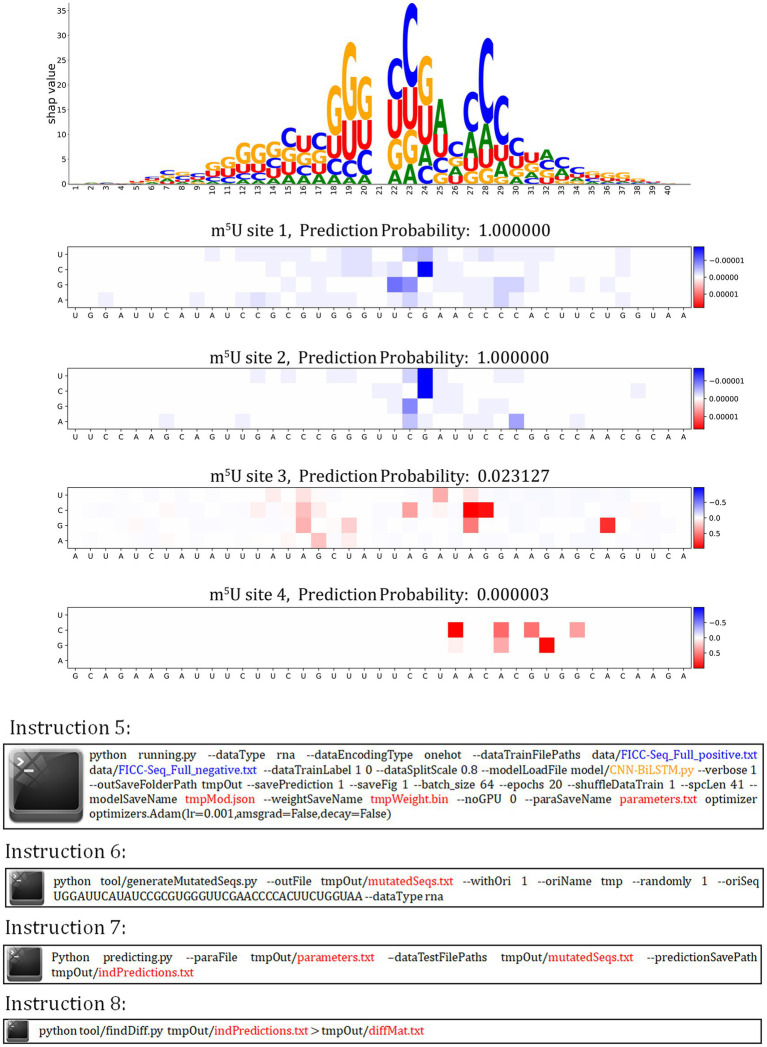
autoBioSeqpy visualization of *in silico* mutagenesis on the CNN-BiLSTM model for four selected m^5^U sequences in the FICC-Seq dataset.

autoBioSeqpy supports variant effect prediction (Figure 5). We first called the instruction 5 to train the CNN-BiLSTM model on the FICC-Seq dataset. After that, we performed *in silico* saturation mutagenesis of four experimentally verified m^5^U sites, using instruction 6 to convert every position in the sequence to every other possible base. We predicted these mutated sequences (instruction 7) and calculated mutation effect scores by measuring the changes in their predicted probabilities (instruction 8). A Jupyter notebook tutorial was provided for showing the details of plot generation.^2^ According to the distribution displayed in the heatmap, we found that for the sites which the CNN-BiLSTM model correctly predicted, the difference values were very small (less than 1e-4), but the sites which did not correctly predicted by the CNN-BiLSTM model, a part of the mutation increased the probability by more than 50%. This observation can be explained by the structure of LSTM structure that few change of a word vector (i.e., the mixture one-hot encoded bases by CNN in this work) will change the hidden states and thus will affect the final decision layer. Based on this property, the region contain high different probabilities can be a reference for further research. At the same time, we also observed that the distribution of high difference probabilities in heat map is similar with the region of large SHAP values, which could support that the region of high difference probabilities contains research value.

### Performance comparison of Deepm5U with the exiting method

We compared Deepm5U’s performance against the recently published algorithm m5UPred, which was that trained and tested on the same benchmark datasets as ours. Deepm5U produced more accurate training classification results (*ACC* scores of 92.32 and 91.47%) for full transcript mode and mature mRNA mode, respectively, than does m5UPred (88.32%, 89.91%; Table 3). We also observed better independent test performance with Deepm5U (*ACC* scores of 92.91% and 92.48%% for full transcript mode and mature mRNA mode) than that of m5UPred (88.35%, 89.70%). For the cross-technique and cross-cell-type evaluations, the comparison results were shown in Supplementary Table S7. In this comparison we see that Deepm5U produced the better performance (miCLIP-Seq *MCC*: 0.7093 and 0.8945, FICC-Seq *MCC*: 0.5574 and 0.6574, HEK293 *MCC*: 0.8135 and 0.9181, HAP1 *MCC*: 0.5967 and 0.6408) compared with m5UPred (miCLIP-Seq *MCC*: 0.6520 and 0.8090, FICC-Seq *MCC*: 0.4950 and 0.4490, HEK293 *MCC*: 0.7260 and 0.8450, HAP1 *MCC*: 0.5070 and 0.4610) for the two modes.

**Table 3:** Performance comparison of Deepm5U and m5UPred on the training and independent test datasets for two modes.

Method	ACC (%)	*F*-value (%)	Recall (%)	PRE (%)	MCC	auROC	auPR
Full transcript (Training dataset)							
m5UPred	88.32	–	87.59	–	0.7670	0.9560	–
Deepm5U	92.32	92.29	91.93	92.65	0.8465	0.9740	0.9781
Full transcript (Independent test set)							
m5UPred	88.35	–	87.90	–	0.7670	0.9560	–
Deepm5U	92.91	92.96	93.79	92.16	0.8584	0.9773	0.9810
Mature mRNA (Training dataset)							
m5UPred	89.91	–	88.64	–	0.7980	0.9560	–
Deepm5U	91.47	91.04	89.83	92.58	0.8304	0.9640	0.9648
Mature mRNA (Independent test set)							
m5UPred	89.70	–	87.44	–	0.7950	0.9540	–
Deepm5U	92.48	92.47	92.65	92.30	0.8498	0.9511	0.9473

## Discussion

RNA chemical modifications can influence biological function. Accurate transcriptome-wide mapping and single-nucleotide resolution detection of these dynamic RNA modifications are critical for understanding gene regulation and function. In recent years, deep learning methods have provided remarkably good results in various biological applications, including the identification of various epitranscriptomic marks. Nevertheless, choosing the best-suited models and proper fine-tuning strategies remains a significant challenge for the development of personalized prediction algorithms based on the user’s data. There is a pressing need to develop user-friendly and model-adjustable environments for building and running DL models.

autoBioSeqpy is our contribution to the field for the accessibility and dissemination of deep learning techniques in biology. The autoBioSeqpy environment facilitates the creation of reproducible workflows and results for developers and end users, reducing the tedious modeling process in the routinely performed biological sequence classification tasks. By leveraging autoBioSeqpy, here we have explored the use of DL methods to identify RNA modifications such as m^5^U methylation. Various common DL model architectures were evaluated, including CNN, BiGRU, BiLSTM, CNN-BiGRU and CNN-BiLSTM. Our systematic and comprehensive benchmark study suggests that deep-learning-based algorithms that rely only on RNA sequence are effective in predicting potential m^5^U sites, outperforming current state-of-the-art tool. In particular, the performance of CNN-BiLSTM model was consistently better than all other DL models and was therefore chosen as the final predictor, called Deepm5U, for subsequent experiments and comparisons. We have also introduced two interpretability approaches to elucidate the mechanism of model and the influence of features. This has explained quite a few interesting phenomena that cannot be uncovered by conventional motif analysis. Furthermore, we have provided a step-by-step guide on how to use autoBioSeqpy to run model development and analysis tasks, and hope that this strategy can be extended to facilitate the study of other RNA modifications.

## Data availability statement

The original contributions presented in the study are included in the article/Supplementary material, further inquiries can be directed to the corresponding authors.

## Author contributions

LY designed the study, collected the samples, and wrote the manuscript. YZ performed the experiments and analyzed the data. LX and FL analyzed the data. RJ designed the study and performed the experiments. JL designed the study, analyzed the data and wrote the manuscript. All authors contributed to the article and approved the submitted version.

## Funding

This work was supported by the National Natural Science Foundation of China (Nos. 22203057) Fund of Science and Technology Department of Guizhou Province ([2017]5790–07), Natural Science Foundation of Department of Education of Guizhou Province ([2021]021), Joint project of Luzhou Municipal People’s Government and Southwest Medical University (2020LZXNYDJ39).

## Conflict of interest

The authors declare that the research was conducted in the absence of any commercial or financial relationships that could be construed as a potential conflict of interest.

## Publisher’s note

All claims expressed in this article are solely those of the authors and do not necessarily represent those of their affiliated organizations, or those of the publisher, the editors and the reviewers. Any product that may be evaluated in this article, or claim that may be made by its manufacturer, is not guaranteed or endorsed by the publisher.

## Footnotes

^1^ https://github.com/jingry/autoBioSeqpy/blob/2.0/notebook/Understanding%20the%20contributions%20from%20the%20inputs%20using%20shaps%20(onehot%20case).ipynb

^2^ https://github.com/jingry/autoBioSeqpy/blob/2.0/notebook/An%20Example%20of%20Mutation%20Plotting.ipynb
