# Supplementary material for: Evaluation and development of deep neural networks for RNA 5-Methyluridine classifications using autoBioSeqpy
Source: Front Microbiol. 2023 May 18;14:1175925. doi: 10.3389/fmicb.2023.1175925 (PMC10232852; doi:10.3389/fmicb.2023.1175925)
Supplement: Supplementary file 1 [file Data_Sheet_1.docx]

**Supplemental Materials**

**Evaluation and development of deep neural networks for RNA 5-Methyluridine classifications using autoBioSeqpy**

Lezheng Yu^1,†^, Yonglin Zhang^2,†^, Li Xue^3^, Fengjuan Liu^4^, Runyu Jing^5^,* and Jiesi Luo^6,7,*^

^1^School of Chemistry and Materials Science, Guizhou Education University, Guiyang, China, ^2^Department of Pharmacy, Affiliated Hospital of North Sichuan Medical College, Nanchong, China, ^3^School of Public Health, Southwest Medical University, Luzhou, China, ^4^School of Geography and Resources, Guizhou Education University, Guiyang, China, ^5^School of Cyber Science and Engineering, Sichuan University, Chengdu, China, ^6^Basic Medical College, Southwest Medical University, Luzhou 646000, China, ^7^Sichuan Key Medical Laboratory of New Drug Discovery and Druggability Evaluation, Luzhou Key Laboratory of Activity Screening and Druggability Evaluation for Chinese Materia Medica, Southwest Medical University, Luzhou 646000, China

***Corrspondence:**

Runyu Jing

jingryedu@gmail.com

Jiesi Luo

ljs@swmu.edu.cn

^†^These authors have contributed equally to this work and share first authorship.

**Supplementary Table S1.** Base datasets used for building our m^5^U site predictor

| Type | Mode | Positive samples | Negative samples | Name |
| --- | --- | --- | --- | --- |
| Training dataset | Full transcript | 2956 | 2956 | *Full_train* |
|  | Mature mRNA | 985 | 985 | *Mature_train* |
| Independent test set | Full transcript | 740 | 740 | *Full_test* |
|  | Mature mRNA | 247 | 247 | *Mature_test* |

**Supplementary Table S2.** Validation datasets used for evaluating the performance of Deepm5U

| Mode | Cross type | Specific type | Testing Method | Positive samples | Negative samples | Name |
| --- | --- | --- | --- | --- | --- | --- |
| Full transcript | Cell line | HAP1 | Cross validation | 1229 | 12290 | *HAP_full* |
|  |  |  | Independent test set | 2467 | 24670 | *HEK_full* |
|  |  | HEK293 | Cross validation | 2467 | 24670 | *HEK_full* |
|  |  |  | Independent test set | 1229 | 12290 | *HAP_full* |
|  | Technique | FICC-Seq | Cross validation | 1471 | 14710 | *FICC_full* |
|  |  |  | Independent test set | 2225 | 22250 | *miCLIP_full* |
|  |  | miCLIP-Seq | Cross validation | 2225 | 22250 | *miCLIP_full* |
|  |  |  | Independent test set | 1471 | 14710 | *FICC_full* |
|  |  |  |  |  |  |  |
| Mature mRNA | Cell line | HAP1 | Cross validation | 364 | 3640 | *HAP_mature* |
|  |  |  | Independent test set | 868 | 8680 | *HEK_mature* |
|  |  | HEK293 | Cross validation | 868 | 8680 | *HEK_mature* |
|  |  |  | Independent test set | 364 | 3640 | *HAP_mature* |
|  | Technique | FICC-Seq | Cross validation | 409 | 4090 | *FICC_mature* |
|  |  |  | Independent test set | 823 | 8230 | *miCLIP_mature* |
|  |  | miCLIP-Seq | Cross validation | 823 | 8230 | *miCLIP_mature* |
|  |  |  | Independent test set | 409 | 4090 | *FICC_mature* |

**Supplementary Table S3.** Hyperparameter optimization of the CNN and CNN+BiLSTM models on the *Full_train* dataset

| Value | Architecture | ACC (%) | *F*-value (%) | Recall (%) | PRE (%) | MCC |
| --- | --- | --- | --- | --- | --- | --- |
| Filter number | | | | | | |
| 50 | CNN | 88.77 | 88.73 | 88.59 | 88.97 | 0.7764 |
| 150 |  | 88.49 | 88.37 | 87.75 | 89.13 | 0.7709 |
| **250** |  | **89.10** | **89.21** | **88.91** | **89.31** | **0.7831** |
| 50 | CNN-BiLSTM | 87.88 | 88.21 | 87.52 | 88.97 | 0.7580 |
| 150 |  | 88.06 | 88.09 | 88.03 | 88.42 | 0.7630 |
| **250** | CNN-BiGRU | **88.84** | **88.75** | **88.41** | **89.20** | **0.7777** |
| 50 |  | 88.42 | 88.52 | 89.93 | 87.22 | 0.7695 |
| 150 |  | 88.86 | 89.03 | 89.36 | 88.75 | 0.7776 |
| **250** |  | **88.98** | **88.90** | **88.48** | **89.47** | **0.7807** |
| Kernel size | | | | | | |
| 3 | CNN | 89.10 | 89.21 | 88.91 | 89.31 | 0.7831 |
| 5 |  | 88.18 | 87.96 | 88.19 | 87.79 | 0.7638 |
| 7 |  | 88.62 | 88.74 | 88.43 | 89.07 | 0.7723 |
| 9 |  | 88.96 | 89.05 | 88.36 | 89.77 | 0.7795 |
| **11** |  | **89.21** | **89.31** | **88.96** | **89.68** | **0.7843** |
| 3 | CNN-BiLSTM | 88.84 | 88.75 | 88.41 | 89.20 | 0.7777 |
| 5 |  | 88.94 | 88.91 | 89.96 | 88.03 | 0.7804 |
| **7** |  | **90.13** | **90.10** | **91.46** | **89.01** | **0.8050** |
| 9 |  | 89.04 | 88.85 | 87.45 | 90.95 | 0.7864 |
| 11 | CNN-BiGRU | 89.89 | 88.85 | 87.45 | 90.95 | 0.7864 |
| 3 |  | 88.98 | 88.90 | 88.48 | 89.47 | 0.7807 |
| 5 |  | 89.74 | 89.56 | 88.49 | 90.74 | 0.7957 |
| 7 |  | 90.28 | 90.32 | 90.92 | 89.76 | 0.8059 |
| 9 |  | 90.89 | 91.06 | 90.86 | 91.29 | 0.8181 |
| **11** |  | **90.94** | **90.98** | **90.59** | **91.44** | **0.8193** |
| Pooling size | | | | | | |
| 2 | CNN | 89.21 | 89.31 | 88.96 | 89.68 | 0.7843 |
| 4 |  | 89.23 | 89.22 | 88.75 | 89.72 | 0.7848 |
| 6 |  | 90.35 | 90.29 | 90.46 | 90.18 | 0.8073 |
| 8 |  | 89.70 | 89.64 | 88.68 | 90.64 | 0.7945 |
| **10** |  | **91.07** | **91.10** | **90.61** | **92.03** | **0.8216** |
| 2 | CNN-BiLSTM | 90.13 | 90.10 | 91.46 | 89.01 | 0.8050 |
| **4** |  | **90.45** | **90.29** | **89.36** | **91.29** | **0.8095** |
| 6 |  | 89.40 | 89.48 | 90.51 | 88.55 | 0.7889 |
| 8 |  | 89.28 | 89.67 | 92.21 | 87.47 | 0.7887 |
| 10 | CNN-BiGRU | 89.08 | 89.42 | 90.81 | 88.14 | 0.7825 |
| 2 |  | 90.94 | 90.98 | 90.59 | 91.44 | 0.8193 |
| 4 |  | 90.96 | 91.04 | 91.32 | 90.78 | 0.8190 |
| 6 |  | 90.96 | 91.08 | 92.24 | 90.00 | 0.8199 |
| 8 |  | 89.25 | 89.09 | 87.88 | 90.33 | 0.7853 |
| **10** |  | **91.21** | **91.23** | **90.35** | **92.16** | **0.8245** |
| Convolution layer number | | | | | | |
| 1 | CNN | 91.07 | 91.10 | 90.61 | 92.03 | 0.8216 |
| **2** |  | **91.56** | **91.50** | **90.04** | **93.04** | **0.8319** |
| 3 |  | 89.15 | 88.77 | 86.84 | 90.98 | 0.7851 |
| 1 | CNN-BiLSTM | 90.45 | 90.29 | 89.36 | 91.29 | 0.8095 |
| **2** |  | **92.32** | **92.29** | **91.93** | **92.65** | **0.8465** |
| 3 | CNN-BiGRU | 90.87 | 91.00 | 91.74 | 90.29 | 0.8176 |
| **1** |  | **91.85** | **91.94** | **92.46** | **91.44** | **0.8370** |
| 2 |  | 90.87 | 90.67 | 90.70 | 90.64 | 0.8173 |
| 3 |  | 89.97 | 89.45 | 87.57 | 91.48 | 0.8002 |

**Supplementary Table S4.** Hyperparameter optimization of the BiLSTM and CNN+BiLSTM models on the *Full_train* dataset

| Value | Architecture | ACC (%) | F-value (%) | Recall (%) | PRE (%) | MCC |
| --- | --- | --- | --- | --- | --- | --- |
| LSTM size | | | | | | |
| 32 | BiLSTM | 78.58 | 82.34 | 84.38 | 80.48 | 0.6412 |
| 64 |  | 83.42 | 83.67 | 86.89 | 80.77 | 0.6714 |
| 128 |  | 84.92 | 84.56 | 81.31 | 88.30 | 0.7019 |
| **256** |  | **88.08** | **87.95** | **87.75** | **88.20** | **0.7620** |
| 32 | CNN+BiLSTM | 89.28 | 89.67 | 92.21 | 87.47 | 0.7887 |
| 64 |  | 90.45 | 90.29 | 89.36 | 91.29 | 0.8095 |
| 128 |  | 89.74 | 89.47 | 89.23 | 89.90 | 0.7958 |
| **256** |  | **90.48** | **90.60** | **89.61** | **91.73** | **0.8108** |
| BiLSTM layer number | | | | | | |
| 1 | BiLSTM | 88.08 | 87.95 | 87.75 | 88.20 | 0.7620 |
| **2** |  | **88.59** | **88.55** | **87.58** | **89.65** | **0.7730** |
| 3 |  | 88.17 | 88.05 | 87.80 | 88.33 | 0.7634 |
| 1 | CNN+BiLSTM | 90.45 | 90.29 | 89.36 | 91.29 | 0.8095 |
| **2** |  | **92.32** | **92.29** | **91.93** | **92.65** | **0.8465** |
| 3 |  | 90.87 | 91.00 | 91.74 | 90.29 | 0.8176 |

**Supplementary Table S5.** Hyperparameter optimization of the BiGRU and CNN+BiGRU models on the *Full_train* dataset

| Value | Architecture | ACC (%) | F-value (%) | Recall (%) | PRE (%) | MCC |
| --- | --- | --- | --- | --- | --- | --- |
| LSTM size | | | | | | |
| 32 | BiGRU | 86.64 | 86.68 | 87.80 | 85.67 | 0.7337 |
| 64 |  | 87.39 | 87.64 | 88.17 | 87.15 | 0.7479 |
| 128 |  | 87.40 | 87.45 | 87.18 | 87.77 | 0.7485 |
| **256** |  | **87.22** | **87.27** | **87.04** | **87.60** | **0.7820** |
| 32 | CNN+BiGRU | 90.99 | 90.98 | 91.25 | 90.72 | 0.8198 |
| 64 |  | 91.21 | 91.23 | 90.35 | 92.16 | 0.8245 |
| 128 |  | 91.85 | 91.69 | 91.85 | 91.53 | 0.8369 |
| **256** |  | **91.85** | **91.94** | **92.46** | **91.44** | **0.8370** |
| BiGRU layer number | | | | | | |
| **1** | BiGRU | **87.22** | **87.27** | **87.04** | **87.60** | **0.7820** |
| 2 |  | 87.90 | 87.66 | 86.87 | 88.53 | 0.7584 |
| 3 |  | 88.20 | 88.17 | 90.01 | 86.44 | 0.7651 |
| **1** | CNN+BiGRU | **91.85** | **91.94** | **92.46** | **91.44** | **0.8370** |
| 2 |  | 90.89 | 90.84 | 90.97 | 90.90 | 0.8191 |
| 3 |  | 90.21 | 90.20 | 91.11 | 89.55 | 0.8067 |

**Supplementary Table S6. Prediction results of the CNN-BiLSTM model on the** **mature mRNA mode**

| Dataset | ACC (%) | *F*-value (%) | | Recall (%) | PRE (%) | MCC |
| --- | --- | --- | --- | --- | --- | --- |
| Training dataset | 91.12 | 91.00 | 91.71 | | 90.31 | 0.8224 |
| Independent test set | 92.07 | 91.75 | 88.57 | | 95.18 | 0.8434 |

**Supplementary Table S7. Prediction results of the CNN-BiLSTM model on the second independent test set**

| Number | Probability | Prediction |
| --- | --- | --- |
| 1 | 0.705720 | 1 |
| 2 | 0.999997 | 1 |
| 3 | 0.724142 | 1 |
| 4 | 0.868581 | 1 |
| 5 | 0.739882 | 1 |
| 6 | 0.999946 | 1 |
| 7 | 0.000108 | 0 |
| 8 | 0.999999 | 1 |
| 9 | 0.999193 | 1 |
| 10 | 1.000000 | 1 |
| 11 | 0.998471 | 1 |
| 12 | 0.993151 | 1 |
| 13 | 0.999161 | 1 |
| 14 | 0.993382 | 1 |
| 15 | 0.990964 | 1 |
| 16 | 0.999985 | 1 |
| 17 | 0.999893 | 1 |
| 18 | 0.001282 | 0 |
| 19 | 1.000000 | 1 |
| 20 | 0.999926 | 1 |
| 21 | 1.000000 | 1 |
| 22 | 1.000000 | 1 |
| 23 | 0.999029 | 1 |
| 24 | 0.999507 | 1 |
| 25 | 0.999663 | 1 |
| 26 | 0.999663 | 1 |
| 27 | 1.000000 | 1 |
| 28 | 0.179193 | 0 |
| 29 | 0.734869 | 1 |
| 30 | 0.284077 | 0 |
| 31 | 0.999486 | 1 |
| 32 | 1.000000 | 1 |
| 33 | 0.512879 | 1 |
| 34 | 0.998790 | 1 |
| 35 | 0.999998 | 1 |
| 36 | 0.992671 | 1 |
| 37 | 0.999999 | 1 |
| 38 | 0.032318 | 0 |
| 39 | 1.000000 | 1 |
| 40 | 0.991149 | 1 |
| 41 | 0.996424 | 1 |

**Supplementary Table S8. Performance comparison of Deepm5U and m5UPred on the Cross-Techniques and Cross-Cell-Type datasets**

| Mode | Testing Method | Method | Evaluation Metric | Cross-Technique Validation | | | Cross-Cell-Type Validation | |
| --- | --- | --- | --- | --- | --- | --- | --- | --- |
|  |  |  |  | miCLIP-Seq | | FICC-Seq | HEK293 | HAP1 |
| Full transcript | intra-dataset evaluation | Deepm5U | *ACC* (%) | 98.31 | 97.41 | | 98.21 | 97.61 |
|  |  | m5UPred |  | 86.76 | 90.58 | | 86.72 | 80.15 |
|  |  | Deepm5U | *Recall* (%) | 87.09 | 79.90 | | 87.01 | 81.50 |
|  |  | m5UPred |  | 86.70 | 89.80 | | 86.26 | 89.67 |
|  |  | Deepm5U | *MCC* | 0.8932 | 0.8362 | | 0.8886 | 0.8478 |
|  |  | m5UPred |  | 0.735 | 0.812 | | 0.735 | 0.901 |
|  |  | Deepm5U | auROC | 0.9769 | 0.9729 | | 0.9858 | 0.9770 |
|  |  | m5UPred |  | 0.946 | 0.966 | | 0.942 | 0.969 |
|  | inter-dataset evaluation | Deepm5U | *ACC* (%) | 95.64 | 93.90 | | 97.10 | 94.33 |
|  |  | m5UPred |  | 82.29 | 73.29 | | 86.20 | 73.99 |
|  |  | Deepm5U | *Recall* (%) | 61.05 | 39.44 | | 74.29 | 44.13 |
|  |  | m5UPred |  | 75.36 | 56.48 | | 82.79 | 57.77 |
|  |  | Deepm5U | *MCC* | 0.7093 | 0.5574 | | 0.8135 | 0.5967 |
|  |  | m5UPred |  | 0.652 | 0.495 | | 0.726 | 0.507 |
|  |  | Deepm5U | auROC | 0.9324 | 0.8332 | | 0.9727 | 0.8700 |
|  |  | m5UPred |  | 0.910 | 0.853 | | 0.941 | 0.857 |
| Mature mRNA | intra-dataset evaluation | Deepm5U | *ACC* (%) | 99.36 | 98.30 | | 99.38 | 98.22 |
|  |  | m5UPred |  | 89.43 | 96.09 | | 90.50 | 96.02 |
|  |  | Deepm5U | *Recall* (%) | 94.62 | 87.80 | | 94.89 | 89.04 |
|  |  | m5UPred |  | 88.34 | 94.14 | | 89.86 | 95.32 |
|  |  | Deepm5U | *MCC* | 0.9621 | 0.8913 | | 0.9619 | 0.8922 |
|  |  | m5UPred |  | 0.789 | 0.922 | | 0.810 | 0.920 |
|  |  | Deepm5U | auROC | 0.9999 | 0.9820 | | 0.9928 | 0.9856 |
|  |  | m5UPred |  | 0.962 | 0.992 | | 0.964 | 0.987 |
|  | inter-dataset evaluation | Deepm5U | *ACC* (%) | 98.28 | 95.06 | | 98.62 | 94.88 |
|  |  | m5UPred |  | 90.46 | 68.16 | | 92.17 | 68.57 |
|  |  | Deepm5U | *Recall* (%) | 89.14 | 48.41 | | 93.90 | 44.77 |
|  |  | m5UPred |  | 90.07 | 38.81 | | 95.41 | 39.01 |
|  |  | Deepm5U | *MCC* | 0.8945 | 0.6574 | | 0.9181 | 0.6408 |
|  |  | m5UPred |  | 0.809 | 0.449 | | 0.845 | 0.461 |
|  |  | Deepm5U | auROC | 0.9931 | 0.8893 | | 0.9943 | 0.8733 |
|  |  | m5UPred |  | 0.970 | 0.873 | | 0.981 | 0.871 |


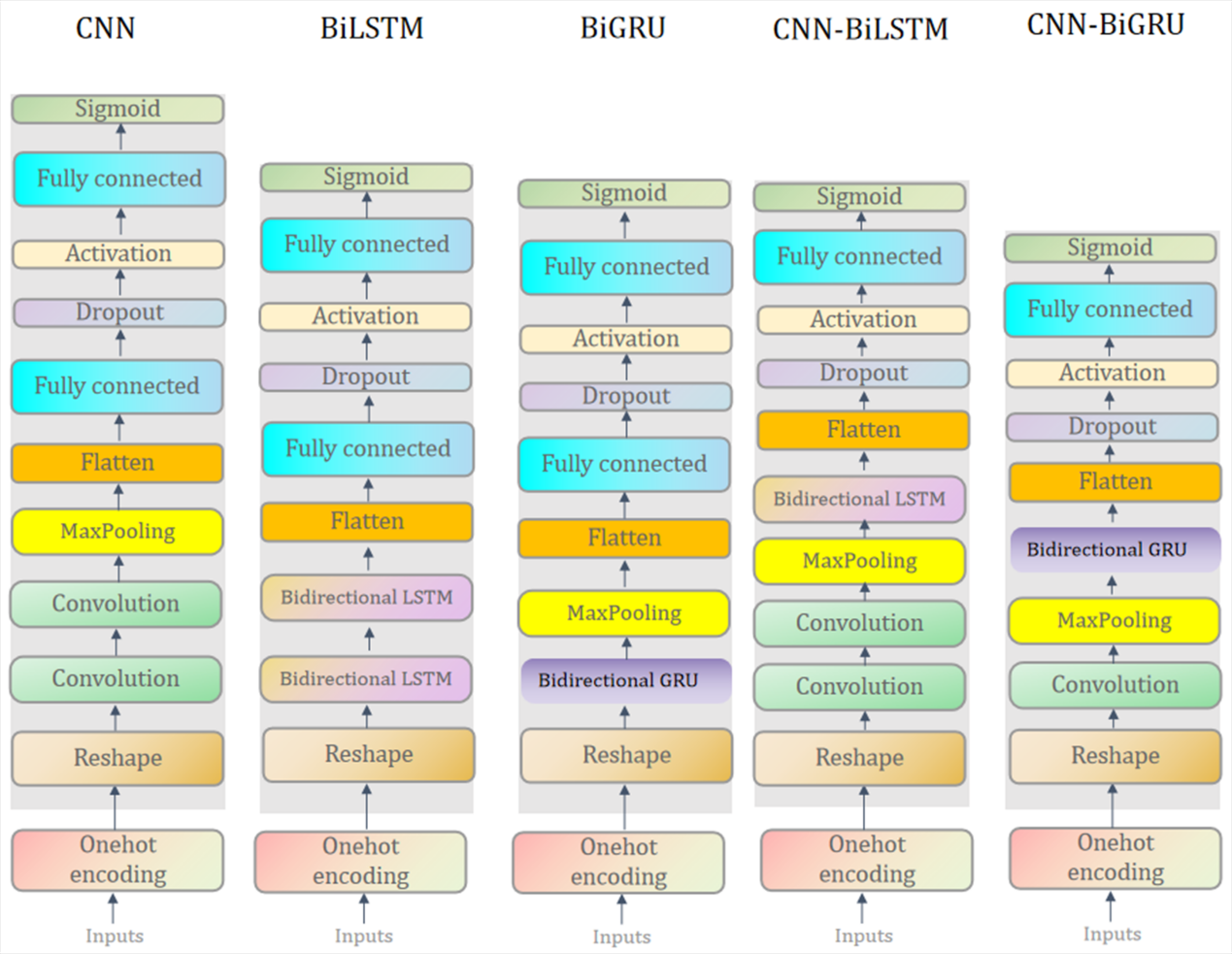


**Supplementary Figure S1**

Workflow of all the deep learning model.


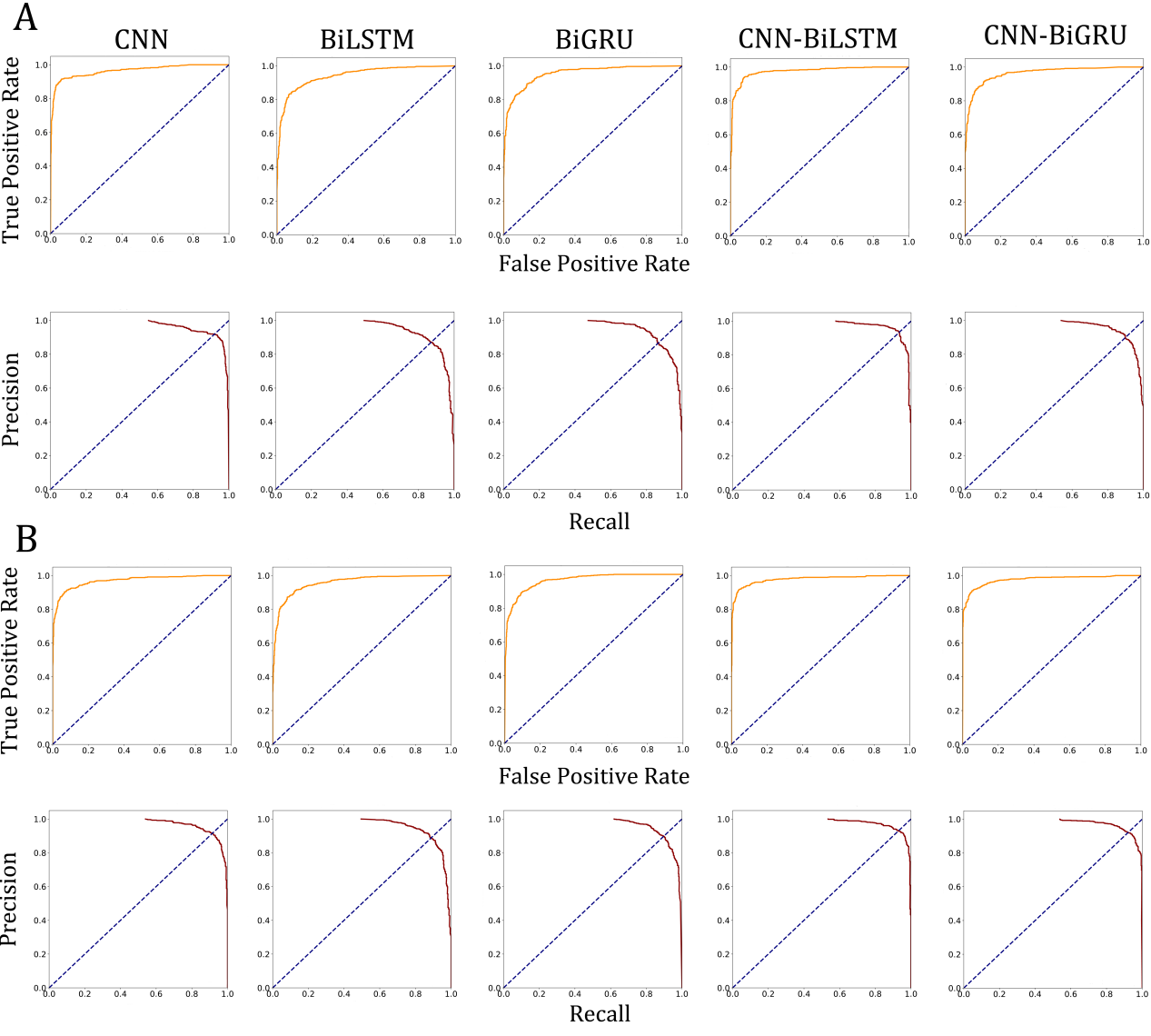


**Supplementary Figure S2**

Receiver operating characteristic (ROC) curves and precision-recall curves (PR) for candidate m5U sites identified by five deep learning models using the set of full transcript mode as ground truth. **(A)** Training dataset, **(B)** Independent test set.


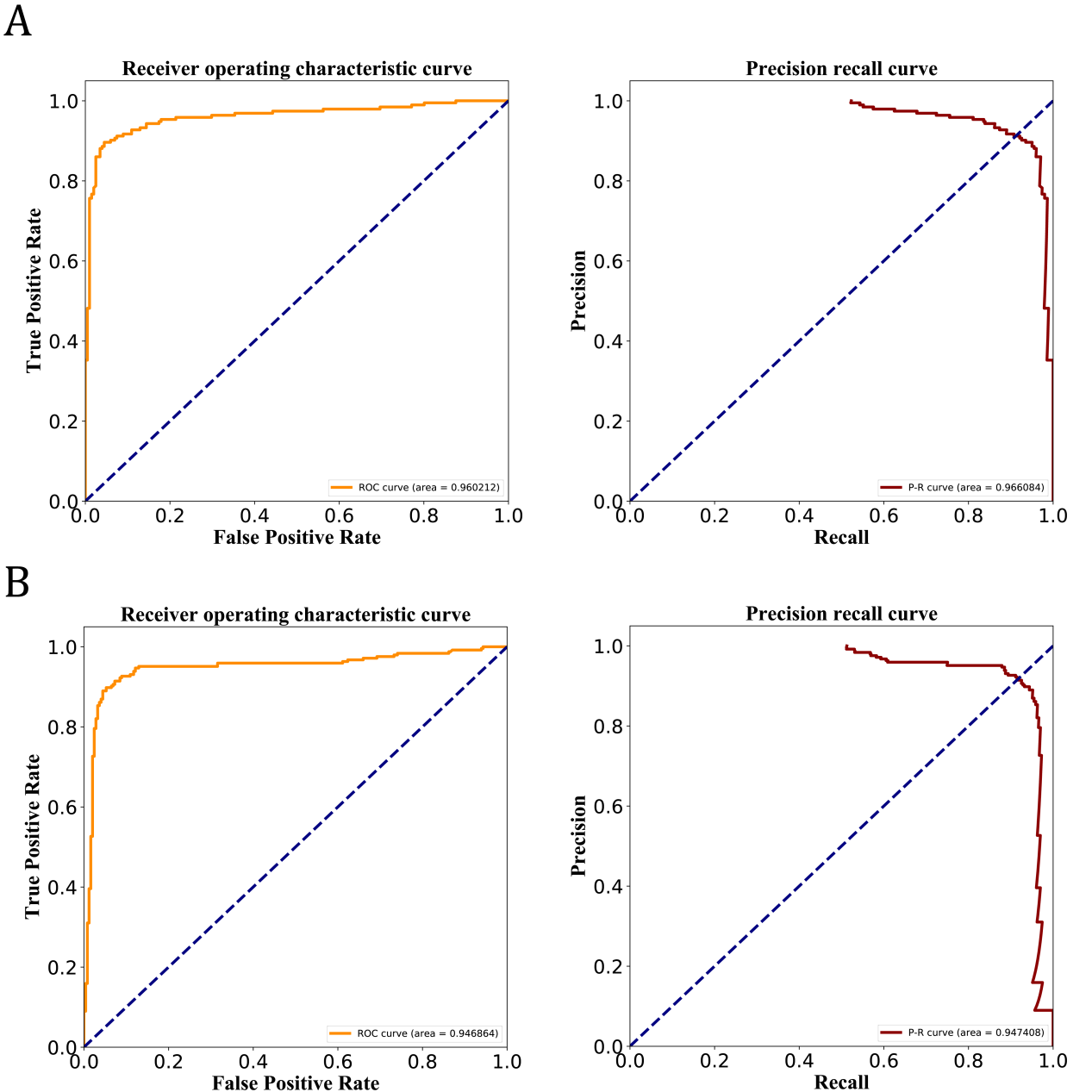


**Supplementary Figure S3**

ROC and PR curves of the CNN-BiLSTM model for the mature mRNA mode. **(A)** Training dataset, **(B)** Independent test set.


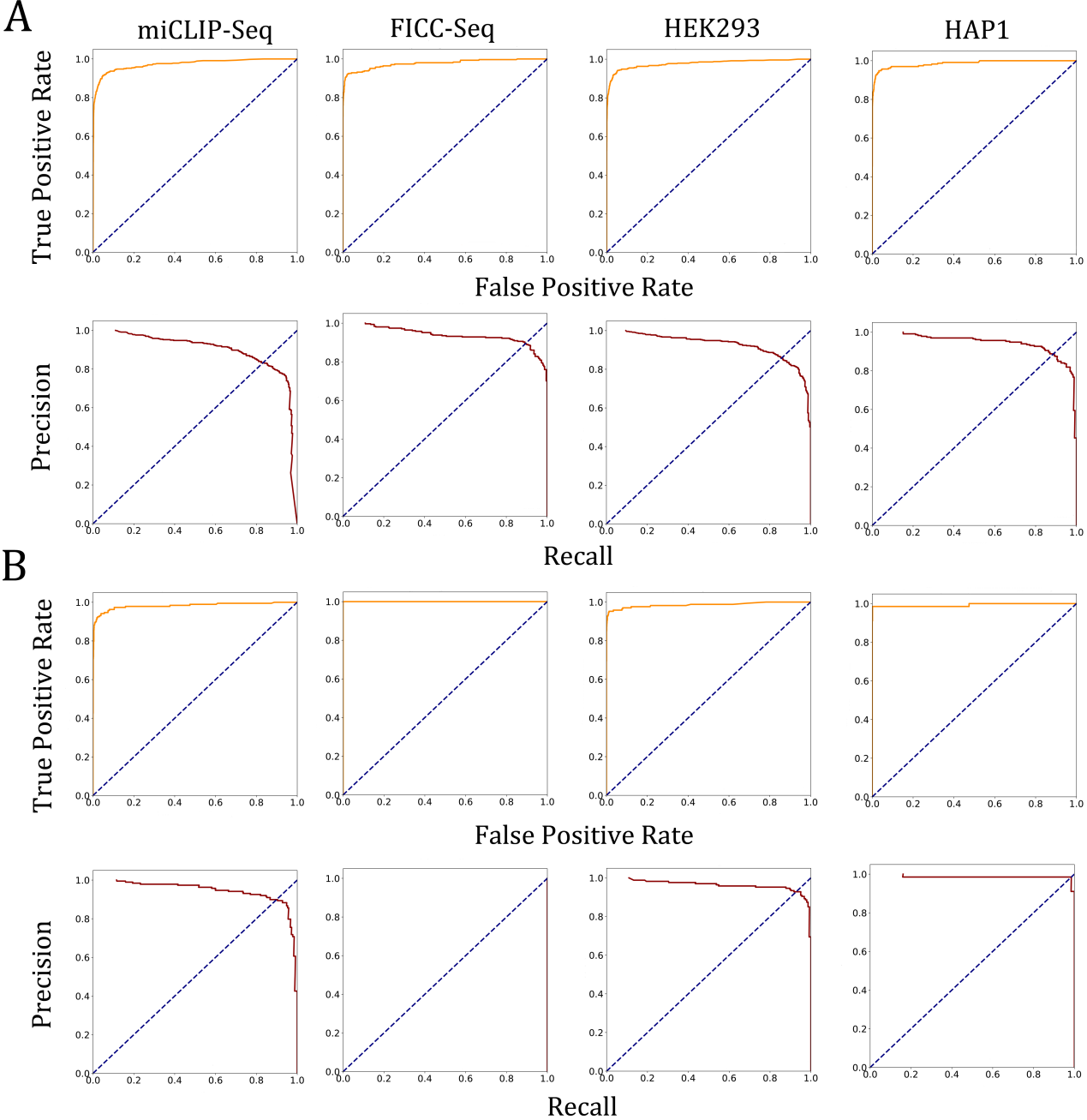


**Supplementary Figure S4**

ROC and PR curves of Deepm5U for the intra-dataset evaluation across technologies and cell types. **(A)** Full transcript mode, **(B)** Mature mRNA mode.


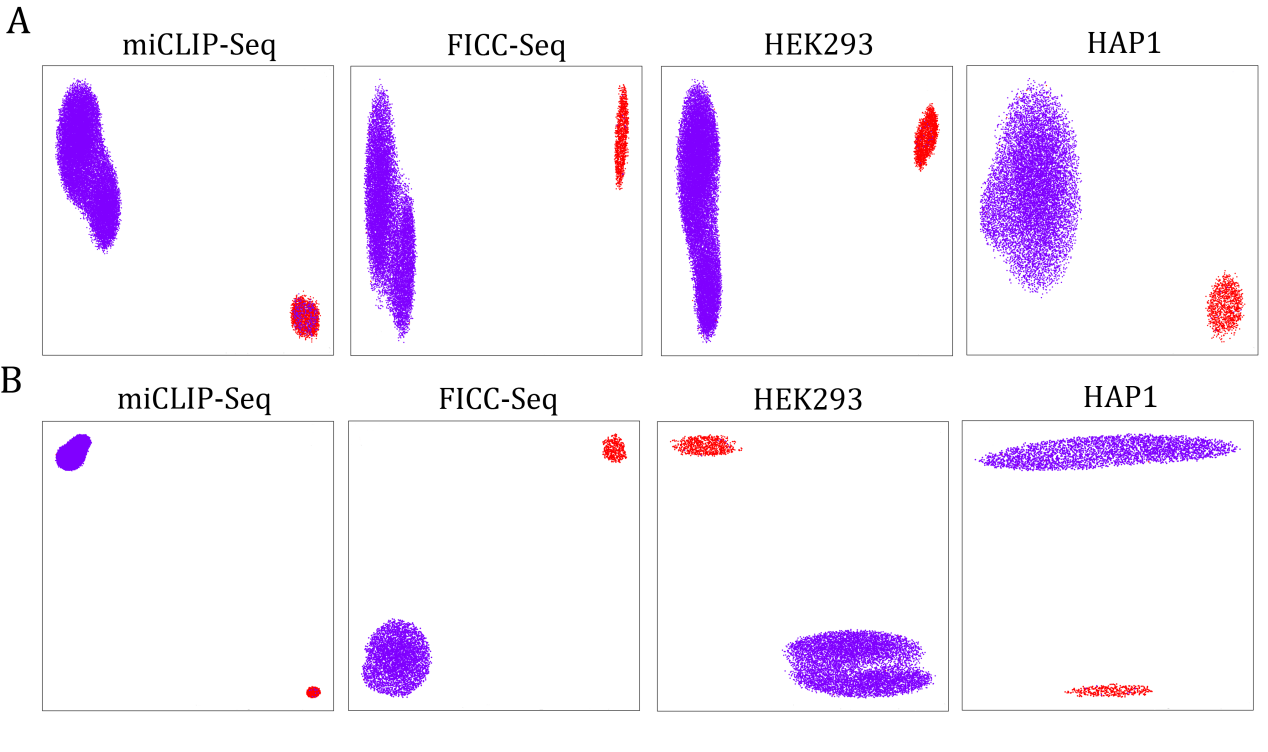


**Supplementary Figure S5**

UMAP visualization of the features learned by Deepm5U from the cross-technique and cross-cell-type datasets.


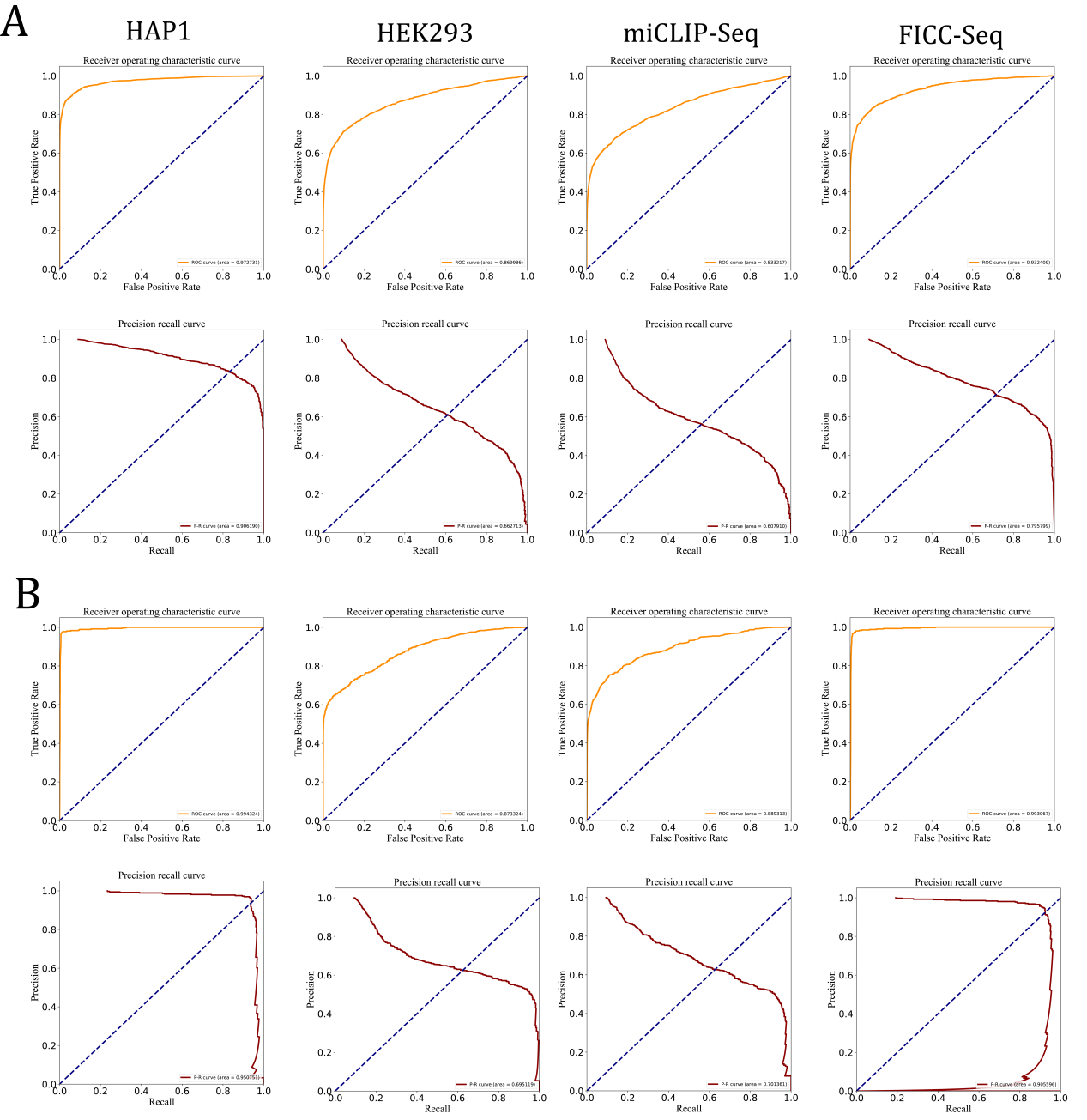
**Supplementary Figure S6**

ROC and PR curves of Deepm5U for the inter-dataset evaluation across technologies and cell types. **(A)** Full transcript mode, **(B)** Mature mRNA mode.
